# RFID Adaptive Parallel Response Collision Tree Algorithm Based on Lock-Bit

**DOI:** 10.3390/s24020389

**Published:** 2024-01-09

**Authors:** Xuan Luo, Xiaolin Jia, Yajun Gu

**Affiliations:** 1 School of Computer Science and Technology, Southwest University of Science and Technology, Mianyang 621010, China; luoxuan@mails.swust.edu.cn (X.L.); guyajun@swust.edu.cn (Y.G.); 2 Mobile Internet of Things and Radio Frequency Identification Technology Key Laboratory of Mianyang (MIOT&RFID), Mianyang 621010, China

**Keywords:** radio frequency identification, collision tree, adaptive strategy, lock-bit, anti-collision

## Abstract

This paper proposes the Lock-Position-Based RFID Adaptive Parallel Collision Tree (LAPCT) algorithm to address the issues of excessive time slots required in the identification process of collision tree algorithms for multiple tags and the high communication complexity between the reader and multiple tags. The LAPCT algorithm adopts a single-query multiple-response mechanism and dynamically divides the response sub-cycle numbers in the identification cycle based on an adaptive strategy. It uses Manchester encoding to lock collision positions and generate a common query prefix, effectively reducing the number of reader queries. This reduction in queries decreases the total number of required time slots and transmitted bits during the reader–tag communication process, thereby improving the efficiency of multiple tag recognition. Theoretical and simulation experiments demonstrate that compared to similar algorithms, the LAPCT algorithm achieves a maximum reduction of 37% in total time slots required, a maximum improvement of 30% in recognition efficiency, and a maximum reduction of 90% in communication complexity. Furthermore, with an increase in the number of tags, the performance advantages of the LAPCT algorithm become more pronounced, making it suitable for large-scale tag scenarios.

## 1. Introduction

The Internet of Things (IoT) endeavors to connect everything, having already facilitated the interconnection and interoperability of billions of devices. The successful proliferation of IoT is closely tied to one of its key technologies—radio frequency identification technology (RFID). RFID is a non-contact wireless communication technology extensively employed in various fields, including logistics management [1], food traceability [2], and commercial retail [3]. An RFID system usually comprises a single reader and multiple tags. However, when multiple tags vie for a shared communication channel to transmit data simultaneously, issues of tag collision may arise. This not only impedes the successful transmission of tag data but also leads to an increase in recognition time and system energy consumption [4]. As the application of the IoT continues to grow, there is a rising prevalence of large-scale labeling scenarios, making the multi-label collision problem more pronounced. Consequently, research on multi-tag recognition technology and anti-collision algorithms has become a core aspect of studies related to RFID technology and applications.

Presently, algorithms designed to tackle tag collision problems can be broadly categorized into two types: ALOHA-based anti-collision algorithms [5] and tree-based anti-collision algorithms [6].

ALOHA-based anti-collision algorithms mainly include the slotted ALOHA algorithm [7], the framed slotted ALOHA algorithm [8], the dynamic framed slotted ALOHA algorithm, and their improved algorithms [9]. These algorithms employ a random access mechanism, which leads to the common issue of “tag starvation” in ALOHA class algorithms [10]. In contrast, tree-based anti-collision algorithms use a tree-like structure to identify tags one by one, effectively addressing the “tag starvation” problem.

Anti-collision algorithms based on tree structures are mainly classified into three categories: query tree algorithms (QT) [11], binary search tree (BS) algorithms [12], and collision tree algorithms (CT) [13]. Among these, CT algorithms utilize Manchester encoding [14] to acquire the first collision bit, generate query prefixes, and eliminate idle slots during the identification process, thereby increasing recognition efficiency by 50%. The Binary Collision Tree algorithm (BCT) [15], based on CT algorithms, introduces a dual-response mechanism following the binary deterministic principle, dividing the RFID identification cycle into two sub-cycles. However, this algorithm still experiences relatively long identification delays due to its binary tree structure. The Adaptive Collision Tree algorithm (ACT) [16] proposes an adaptive mechanism to dynamically adjust the collision tree’s branch structure. The Parallel-Matching Adaptive Collision Tree algorithm (PACT) [17] introduces a parallel matching mechanism to reduce system time complexity, although its communication complexity remains high. The Collision Tree Window algorithm (CwT) [18], based on CT algorithms, suggests a heuristic bit window strategy to conserve the reader’s energy cost in the RFID system, effectively reducing the message bit count for tag transmissions [19]. However, it increases the system cost for each query command. The Dynamic Collision Tree algorithm (DCT) [20], designed for dynamic RFID systems, tracks collision bits in response tags and correctly identifies them. The Multibranch Collision Tree algorithm (MCT) [21] locks the first log_2_M collision bits in tag responses and iteratively queries collision tags in M sub-cycles. However, a fixed M value can result in many idle sub-cycles, reducing system efficiency.

In summary, to tackle the challenges posed by the excessive total number of slots and transmitted bits in many collision tree-based algorithms, this paper proposes the Lock-Bit-Based RFID Adaptive Parallel Response Collision Tree algorithm (LAPCT). The key contributions are as follows:

- Adopting a parallel response mechanism to replace the traditional reader-to-tag response mechanism in RFID systems, aiming to reduce the number of transmitted bits when the reader sends a query command during communication.
- Generating a common query prefix by utilizing Manchester encoding to lock collision bits, simplifying the reader’s query commands.
- Adaptively selecting the number of response cycles based on search depth to reduce the number of idle response sub-cycles.
- Theoretical analysis and simulation experiments demonstrate that the LPACT algorithm outperforms similar algorithms such as ACT, MCT, and PACT in terms of the total number of time slots required by the system, the number of transmitted bits in the communication process between the reader and tags, and the system identification efficiency.

## 2. Materials and Methods

### 2.1. Parallel Response Model

**Figure 1:**
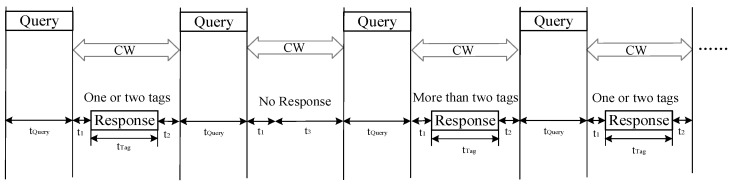
Reader and tag query–response communication model in traditional tree algorithms.

The query–response communication model between readers and tags in traditional tree-based algorithms adheres to the EPC C1 Gen2 standard [22], as illustrated in Figure 1. In the query phase, the reader transmits a query command, and during the tag recognition process, the reader emits a carrier wave (CW) to power the tag, enabling the tag to respond with its ID information. Tags undergo three states during the recognition process: a transmission state, a waiting state, and a sleep state. Specifically, when the tag’s ID matches the query prefix sent by the reader, it enters the transmission state, responding to the reader with its ID information. After the reader successfully reads this tag’s information, it transitions to the sleep state. Conversely, if the tag’s ID does not match the query prefix sent by the reader, the tag remains in the waiting state.

**Figure 2:**
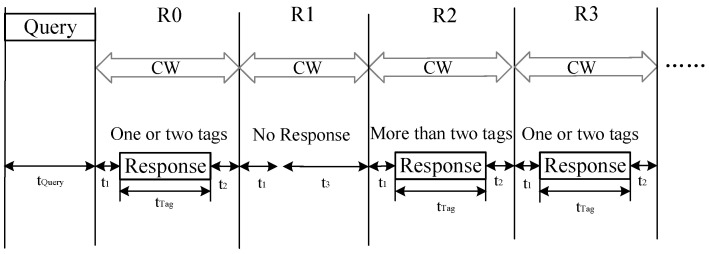
Query–response communication model between reader and tag in the LPACT algorithm.

In the LAPCT algorithm, the query–response communication model between the reader and tags is depicted in Figure 2. Following the transmission of a query command by the reader, the tag response period during tag recognition is segmented into multiple response sub-cycles. Tags matching the query command respond to the reader in different response sub-cycles. In the LPACT algorithm, this communication model is denoted as the parallel response model. For instance, with the number of sub-response periods set to 4, *R*0~*R*3 represent four response sub-cycles. In this mode of communication, the reader can eliminate three unnecessary queries.

### 2.2. Adaptive Strategy

In the context of RFID arbitration trees [23], it is proposed that the fewer branches there are in a multibranch tree, the more collision slots there are, whereas the more branches there are, the more idle slots are available. Similarly, when it comes to the number of response sub-cycles, fewer sub-cycles result in more collision slots, while more sub-cycles lead to more idle slots. Therefore, in an RFID system, if collisions are infrequent and the system load is high, reducing the number of branches can decrease collision conflicts. Conversely, if collisions are severe and the system load is low, increasing the number of branches can improve throughput. This strategy can be adjusted based on specific circumstances to optimize the performance of the RFID system to the maximum extent.

Assuming that there are *N* tags to be identified in the RFID field, with a search depth of *k* and a reader sending query commands, the probability of tag recognition *P*(*k*) can be described as [17]:

(1) $$P(k)=p(1){\left[1-p(1)\right]}^{k-1}$$

(2) $$p(1)={\left(1-\frac{1}{j}\right)}^{N-1}$$

Substituting Equation (2) into Equation (1), the expected value of the search depth *k* can be obtained as:

(3) $$E(k)={\sum_{k=1}^{\infty }kP(k)=(1-\frac{1}{j})}^{1-N}$$

When the reader deals with a single collision, i.e., when *j* = 2, the average identification slot count can be represented as:

(4) $$\bar{T_{\left(R=2\right)}}=E(k)*j=\frac{2}{{\left(1-1/2\right)}^{N-1}}$$

When the reader handles two collisions, i.e., when *R* = 4, the average identification slot count can be represented as:

(5) $$\bar{T_{\left(R=4\right)}}=E(k)*j=\frac{4}{{\left(1-1/4\right)}^{N-1}}$$

From Equations (4) and (5), it can be observed that when the value of *N* is less than 3, the value of *j* is set to 2, resulting in fewer required average identification slots. However, when the value of *N* is greater than or equal to 3, the value of *j* is set to 4, leading to even fewer required average slots. Therefore, an adaptive strategy is employed to dynamically adjust the value of *j* (*j* = 2 or *j* = 4) in the LPACT algorithm. This means that the LPACT algorithm selects either a dual-response sub-cycle or a quad-response sub-cycle to generate common prefixes for querying tags within the reader’s radio frequency identification range.

Assuming that the average number of tags in each collision slot is 3, when the search depth is greater than *k*, the reader processes a collision bit and divides the response period into two response sub-cycles, *R*0 and *R*1. When the search depth is less than *k*, the reader processes two collision bits and divides the response sub-cycles into four response sub-cycles, *R*0–*R*3, where $k=\left\lfloor \log_{4}N/3\right\rfloor$.

**Figure 3:**
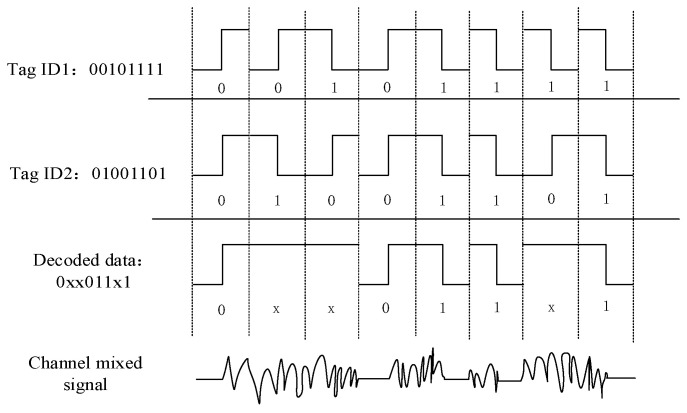
The schematic diagram of the Manchester-coded locking collision bit.

The LAPCT algorithm, like similar collision tree algorithms, uses Manchester encoding to obtain correct data bit positions for responding tags and collision bit position information. The schematic diagram of Manchester coded locking collision bit is shown in Figure 3. When two labels with tag ID “00101111” and tag ID ” 01001101” are transmitted in the wireless channel at the same time, a logic 0 is coded by a positive transition and a logic 1 is coded by a negative transition. The ‘no transition’ state is not permissible during data transmission and is recognized as an error [14]. Therefore, the data obtained by the reader decoded in Figure 3 is “0xx011x1”, where “x” indicates that the data bit cannot be parsed. So, according to the principle of Manchester coding, we can obtain the data bits that have collided as the second, third, and seventh bits.

The collision bit information is used as feature bit information, allowing tags with different feature bits to respond to the reader’s query in different response sub-cycles. The query prefixes stored in the query stack are common query prefixes, meaning that only one query needs to be sent, and tags matching the feature bit information will respond in different response sub-cycles without the need to generate additional query prefixes. Therefore, to obtain the search depth, a counter register needs to be set in the reader. Each time the reader sends a query command, the counter value is incremented, thus increasing the search depth. From this, the reader employs an adaptive strategy to dynamically determine the number of response sub-cycles. When selecting a response sub-cycle number, such as *j* = 2, tags with feature bit information 0 respond in *R*0, while tags with feature bit information 1 respond in *R*1. For a response sub-cycle number of *j* = 4, tags with feature bit information 00 respond in *R*0, those with feature bit information 01 respond in *R*1, tags with feature bit information 10 respond in *R*2, and tags with feature bit information 11 respond in *R*3. The association between tag characteristic bit information and response sub-cycle is illustrated in Table 1.

## 3. Process and Example the LAPCT Algorithm

### 3.1. Process of the LAPCT Algorithm

**Figure 4:**
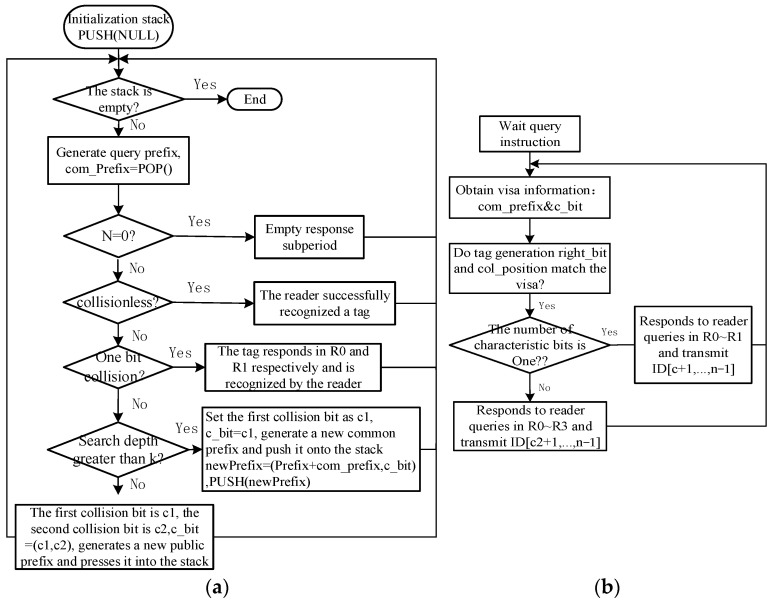
Flow chart of LAPCT algorithm: (**a**) flow chart of reader; (**b**) flow chart of tags.

The reader’s operation process is depicted in Figure 4a and is as follows: firstly, the query stack is initialized. Then, based on the tag response, a query prefix is generated and subsequently sent for querying. If there is no tag response, an empty response sub-cycle is generated. In the case of multiple tag responses resulting in collisions, when the number of collided tags is less than three, the reader uses Manchester encoding to extract the initial collision from the tag response bit string. According to the binary deterministic principle, the response cycle is divided into *R*0 and *R*1, where the collided tags respond in *R*0 and *R*1, respectively. Conversely, the reader extracts the first two collisions from the tag response bit string and records their positions as c1 and c2. The response bit string with c1 and c2 bits set to 1 is represented as com_prefix, with a length of c2 + 1. The position information of the feature bits is represented as c_bit = (c1,c2). The new prefix generation rule is: newPrefix = (Prefix + com_prefix, c_bit). For example, if the reader receives a tag response information of 110x10x, then com_prefix = 1101101, c_bit = (4,7).

The tag operation process is shown in Figure 4b and is as follows: when a tag receives a query command from the reader, the tag extracts the credential information from the query command, namely right_bit and col_position. Here, right_bit represents the correct bit information remaining in the tag response bit string after excluding the collision bits, and col_position represents the position information of the correct bits remaining in the tag response bit string after excluding the collision bits. For instance, if the credential information in the query command is com_prefix = 1101101, c_bit = (4,7), then the matching tag generates right_bit = 11010 and col_position = (1:3,5:6). Therefore, if the tag’s right_bit and col_position information matches the credential information, the tag selects the appropriate response sub-cycle based on the value of the response bits, as described in Table 1.

### 3.2. Example of LAPCT Algorithm

**Figure 5:**
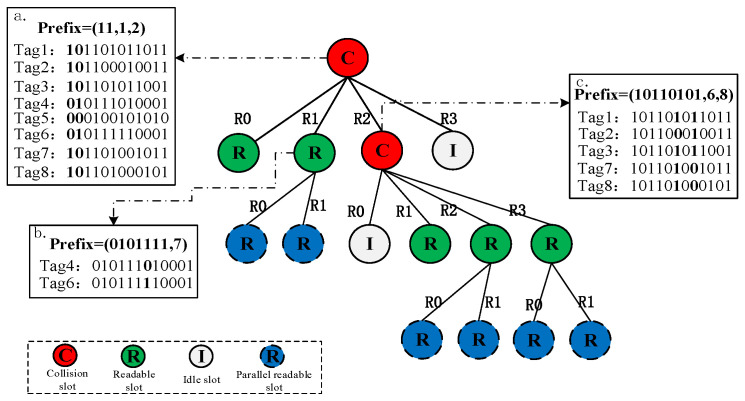
Example diagram of multi-label recognition by LAPCT algorithm.

The process of identifying the tag group Tag1~Tag8 (101101011011, 101100010011, 101101011001, 010111010001, 000100101010, 011111101001, 101101001011, 101101000101) using the LAPCT algorithm is illustrated in Figure 4. The reader first generates a query command based on the tag responses, as shown in box ‘a’. Here, com_prefix = 11 indicates that the reader is handling a collision of two bits, with the position information of the feature bits as c_bit = (1,2). At this point, the reader divides the recognition cycle into four response sub-cycles. The tags extract the query command to obtain information about right_bit and col_position. In this instance, Tag5 with feature bit information 00 responds in the *R*0 sub-cycle, while Tags 4 and 6 with feature bit information 01 employ the binary deterministic dual-response mechanism [17] to respond concurrently in the *R*1 sub-cycle, as shown in box ‘b’ of Figure 4. In box ‘c’ of Figure 5, it can be observed that the tags with feature bit information equal to 10, namely Tag1, Tag2, Tag3, Tag7, and Tag8, respond in Reader 2 (*R*2). At this point, the newly generated common query prefix is com_Prefix = 10110101, with c_bit = (6,8).The tags then extract the query command based on the credential information com_prefix and c_bit. Tags matching the query command respond in their corresponding recognition sub-cycles. Therefore, the reader only needs to send two common prefixes for querying to identify this tag group.

## 4. Performance Analysis of LAPCT Algorithm

To evaluate the performance of the RFID system, we theoretically derived and analyzed three aspects of the LAPCT algorithm: the time complexity, the identification efficiency, and the communication complexity.

### 4.1. Time Complexity of LAPCT

Assuming that there are *N* tags to be identified within the RF field, and the reader has a search depth of *k*, the number of response sub-cycles for tags after the reader sends a query command is denoted as *j.*

When the reader handles two feature bits, *j* = 4. The four response sub-cycles are labeled as *Rj* (*j* = 0, 1, 2, 3), each designated to accommodate tags with feature bit positions of 00, 01, 10, and 11, respectively. In this scenario, there are two cases:

- Feature bit information is 00 and 11.
- Feature bit information is 01 and 10.

Based on this, it can be concluded that in *R*0 and *R*3, there is at least one tag, denoted as “*A*”, and in *R*1 and *R*2, there is at least one tag, denoted as “*B*”, where A=(R0≥1)∩(R3≥1), B=(R1≥1)∩(R2≥1).

Thus, the probability of a response when there are *i* labels in *j* response sub-cycles is expressed as [21].

(6) $$P(j=i|A\cup B)=\frac{P(j=i|A\cup B)*P(j=i)}{P(A\cup B)}=1-2{\left(\frac{1}{3}\right)}^{N-i}$$

Then, when there are four response subcycles, the total number of slots required is

(7) $$S(N|j=4)=\sum_{j=0}^{3}\sum_{i=0}^{i*\left\lfloor \log_{2}N/3\right\rfloor }i*P(j=i|A\cup B)=\left[1-2{\left(\frac{1}{3}\right)}^{N-i}\right]*i$$

After the reader sends a query command, if the search depth is greater than *k*, meaning the reader processes one feature bit, with *j* = 2, the recognition cycle is divided into two response sub-cycles, *R*0 and *R*1. In this state, the total number of required slots is equal to the number of collision slots [13], which can be represented as:

(8) S(N|j=2)=N−1

In summary, the total system slot number of LAPCT algorithm is

(9) $$S(N)=S(N|j=2)+S(N|j=4)=N+\left[1-2{\left(\frac{1}{3}\right)}^{N-i}\right]*i-1$$

According to Section 3 of this paper, the number of queries sent by the reader is actually the number of root nodes in the collision tree structure, so we can obtain the total number of queries m of the reader, described as

(10) $$m=S(N|j=2)/2+S(N|j=4)/4=\frac{\left(2N-1\right)}{2}+\frac{\left[1-2{\left(\frac{1}{3}\right)}^{N-i}\right]*i}{4}$$

In the parallel response model depicted in Figure 2, the time required for the reader to recognize *n* tags is the sum of the time it takes for the reader to send the query instruction and the time needed for the tags to respond, as shown in Formula (11).

(11) $$t(n)=\sum_{a=0}^{m}t_{Query}(a)+\sum_{b=0}^{N(idle)}\left(t_{1}+t_{3}\right)+\sum_{c=0}^{N(succeed)+N(collised)}\left(t_{1}+t_{Tag}+t_{2}\right)$$

where

- *t_Query_*(*a*) indicates the time required by the reader to send instructions in the round a query (the time required by different query instructions varies);
- *N*(*idle*) is the number of empty response subcycles;
- *N*(*succeed*) is the number of successful subcycles;
- *N*(*collised*) are the number of colliding subcycles;
- *S*(*N*) is the total number of subcycles;
- *N*(*idle*), *N*(*succeed*), and *N*(*collided*) add up to *S*(*N*).

### 4.2. Identification Efficiency of LAPCT

The identification efficiency is the ratio of the quantity value of the tags to the total number of slots required to identify these tags [11,13,24]. The identification efficiency can be described as

(12) $$e=\frac{N}{S(N)}=\frac{N}{N+\left[1-2{\left(\frac{1}{3}\right)}^{N-i}\right]*i-1}$$

### 4.3. Communication Complexity of LAPCT

The communication complexity in an RFID system typically refers to the number of bits transmitted during the communication process between the reader and the tags [13,15]. The more bits transmitted, the higher the energy consumption of the system. The communication complexity can be further divided into two aspects: the communication complexity of the reader and the communication complexity of the tag.

Assume that the number of tags in the reader’s recognition range is n. Let *C*(*n*) be the total number of transmitted bits of LPACT, *C_r_*(*n*) be the number of transmitted bits of reader in LAPCT algorithm, and *C_t_*(*n*) be the number of transmitted bits of tag in LAPCT algorithm. The relationship between them can be expressed as:

(13) $$C(n)=C_{r}(n)+C_{t}(n)$$

Firstly, the number of transmitted bits of reader of LAPCT algorithm is analyzed. In the LAPCT algorithm, the multi-label recognition process is divided into two stages—that is, two response cycles and four response cycles.

When the tag response mode is double response, the length of the command word when the reader sends the query command is *L_c_*, and the length of the query prefix transmitted by the reader during the m query is *L_p_*. Then, the number of transmitted bits of the reader of LAPCT *C_r_*(*n*) can be expressed as [13]:

(14) $$C_{r}(n)=\sum_{m=0}^{S(n|j=4)}\left(L_{c}+L_{p}\right)$$

where *S*(*n*|*R* = 4) is the number of slots required when *n* labels are identified and four response cycles are adopted.

Regarding the number of transmitted bits of the tag of the LAPCT algorithm, it is assumed that the length of the bit string transmitted by the tag in response to the reader query request at the m recognition cycle is *L_r_*. Then, in four response cycles, the label communication complex *C_t_*(*n*) of the LAPCT algorithm can be expressed as

(15) $$C_{t}(n)=\sum_{j=0}^{3}\sum_{m=0}^{S(n|j=4)}L_{r}*P(j=i|A\cup B)=\sum_{j=0}^{3}\sum_{m=0}^{S(n|j=4)}L_{r}*\left[1-2{\left(\frac{1}{3}\right)}^{N-i}\right]*i$$

By substituting Equations (14) and (15) into Equation (13), the total number of transmitted bits of the tag of the LAPCT algorithm can be obtained as

(16) $$C(n)=C_{r}(n)+C_{t}(n)=\sum_{m=0}^{S(n|j=4)}\left(L_{c}+L_{p}\right)+\sum_{j=0}^{3}\sum_{m=0}^{S(n|j=4)}L_{r}*\left[1-2{\left(\frac{1}{3}\right)}^{N-i}\right]*i$$

According to the identification process when the LAPCT algorithm adopts four response cycles, the relationship between the length of the label ID *L_id_* and *L_p_* and *L_r_* is expressed as

(17) $$L_{id}=L_{P}+L_{r}-4$$

Similarly, when the tag response mode is four-cycle response, its number of transmitted bits of the tag *C*(*n*) is represented by

(18) $$C_{2}(n)=\sum_{i=1}^{n-1}\left(L_{c(2)}+L_{p(2)}\right)+2\sum_{i=1}^{n-1}L_{r}$$

Moreover, the relationship between the length of the label ID, *L_id_*, and *L_p_* and *L_r_* is satisfied as follows:

(19) $$L_{id}=L_{P}+L_{r}-2$$

Therefore, the total number of transmitted bits of the tag of the LAPCT algorithm is

(20) $$\begin{array}{l} C(n)=C_{j=2}(n)+C_{j=4}(n)\\ =\sum_{m=0}^{S(n|j=4)}\left(L_{c}+L_{p}\right)+\sum_{j=0}^{3}\sum_{m=0}^{S(n|j=4)}L_{r}*\left[1-2{\left(\frac{1}{3}\right)}^{N-i}\right]*i+\sum_{i=1}^{n-1}\left(L_{c(2)}+L_{p(2)}\right)+2\sum_{i=1}^{n-1}L_{r} \end{array}$$

Compared with the MCT algorithm, which selects a fixed number of response periods to identify multiple labels, the LAPCT algorithm dynamically selects the number of response slots according to the adaptive strategy, which reduces the number of idle slots. At the same time, compared with the ACT algorithm, which generates the query prefix according to the first collision position when the collision factor is greater than 0.75, the LAPCT algorithm adopts a bit locking mechanism to extract the collision bit and generate the query prefix, which reduces the number of slots required in the label recognition process and the number of transmitted bits. Therefore, the LAPCT algorithm is superior to the ACT algorithm and the MCT algorithm regarding the total slot number, recognition efficiency and the number of transmitted bits of multi-label recognition.

## 5. Experimental Analysis

We evaluated the performance of the proposed LAPCT protocol and compared it with some relevant benchmark protocols.

In the simulation, we considered a static RFID system comprising a single reader and a large number of passive tags. Adhering to the ISO18000-6 standard [22], the channel data rate was 40 Kbps, and the communication frequency between the reader and the tag was 960 MHz. Electronic labels with a number length of 96 bits were randomly generated, and the labels were evenly distributed, and the number of tags was incrementally varied from 200 to 2000. The LPACT algorithm proposed in this paper was compared to the MCT algorithm, ACT algorithm, and PACT algorithm based on three aspects: time complexity, identification efficiency, and communication complexity through simulation and analysis.

**Figure 6:**
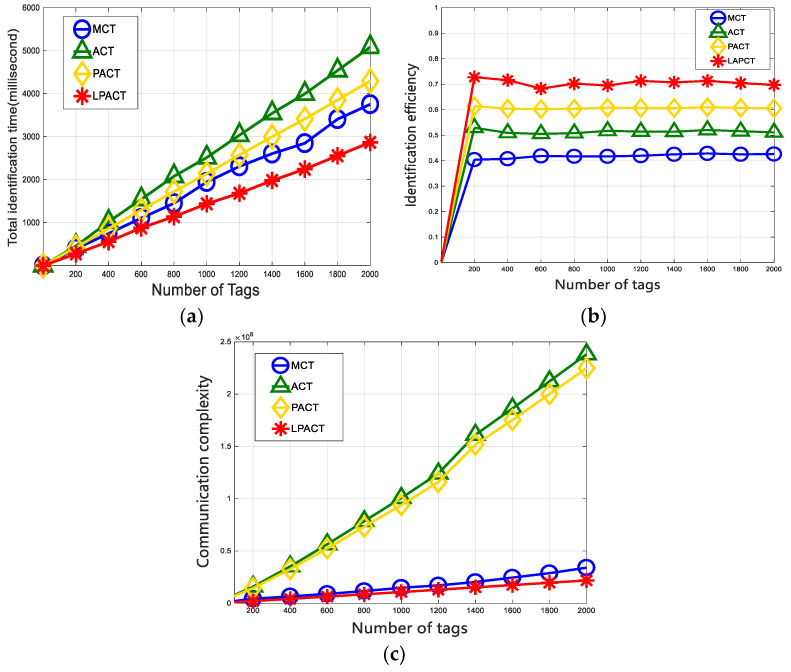
Performance comparison between various algorithms: (**a**) number of total slots; (**b**) system efficiency; (**c**) number of transmitted bits.

The comparison of the LAPCT algorithm with the MCT, ACT, and PACT algorithms in terms of total identification time is depicted in Figure 6a. The graph indicates that, as the number of tags increases, the LAPCT algorithm exhibits a significant advantage in total identification time, necessitating less time compared to the other algorithms.

Figure 6b compares the LAPCT algorithm with the MCT algorithm, ACT algorithm, and PACT algorithm in terms of identification efficiency. From Figure 6, it can be observed that the identification efficiency of the LAPCT algorithm is around 0.7. The PACT algorithm uses a parallel matching mechanism, but its prefix generation method still involves adding slot numbers after collision bits, resulting in an identification efficiency of around 0.6. ACT, which uses a quad-tree search, increases the generation of idle slots in its prefix generation method, leading to an identification efficiency of around 0.5. On the other hand, the MCT algorithm has a fixed number of response cycles, resulting in an identification efficiency of around 0.4. In comparison, the LAPCT algorithm achieves the highest improvement in identification efficiency, up to 30%, when compared to the other three algorithms.

Figure 6c shows the comparison of the LAPCT algorithm with the MCT algorithm, ACT algorithm and PACT algorithm in terms of the number of transmitted bits. As can be seen from Figure 5c, the number of transmitted bits of the LAPCT algorithm and MCT algorithm is much less than that of the ACT algorithm and PACT algorithm. This is because the MCT algorithm and the LAPCT algorithm use Manchester coding to lock collision bits to generate common query prefixes, reduce query times and reduce the communication complexity. In addition, the LAPCT algorithm adopts an adaptive strategy to dynamically select the collision bits for processing, which reduces the number of hollow response sub-cycles of MCT algorithm. Therefore, the number of transmitted bits of the LAPCT algorithm is better than that of the MCT algorithm.

## 6. Conclusions

To address the challenges arising from an excess of slots generated by the collision tree algorithm and its improved version during the process of identifying multiple tags, as well as to mitigate the challenge of high communication complexity, this paper proposed an RFID adaptive parallel response collision tree (LAPCT) algorithm based on lock-bit. The LAPCT algorithm uses a single-query and multi-cycle parallel response mechanism, dynamically selects the number of response sub-cycles according to the adaptive strategy, and uses Manchester encoding to lock collision bits and generate a common query prefix to reduce the number of reader queries and the bits transmitted during the communication between readers and tags. According to theoretical and simulation experiments, compared with the ACT, MCT and PACT algorithms, the LAPCT algorithm not only reduces the total number of slots required by the system and the number of transmitted bits, but also improves the system identification efficiency. As the number of tags increases, the performance advantage becomes more pronounced, making it well-suited to large-scale labeling scenarios. Future research directions may explore the reader and tag recognition issues in dynamic scenes. Additionally, it is worth noting that, currently, the advantages of our proposed algorithm have only been validated through simulation platforms. Experimental verification in real RFID systems will be considered in subsequent studies.

## Figures and Tables

**Table 1:** Mapping relationship between tag characteristic bit information and response sub-cycle.

Tag Characteristic Bit	Response Sub-Cycle
0	*R*0
1	*R*1
00	*R*0
01	*R*1
10	*R*2
11	*R*3

## Data Availability

Data are contained within the article.
